# Cerebral Cortical Activity During Academic Stress Amongst Undergraduate Medical Students at Kampala International University (Uganda)

**DOI:** 10.3389/fpsyt.2022.551508

**Published:** 2022-06-10

**Authors:** Regan Mujinya, Muhamudu Kalange, Juma John Ochieng, Herbert Izo Ninsiima, Ejike Daniel Eze, Adam Moyosore Afodun, Ritah Nabirumbi, Sheu Oluwadare Sulaiman, Emmanuel Kairania, Isaac Echoru, Alfred Omachonu Okpanachi, Kevin Matama, Oscar Hilary Asiimwe, Grace Nambuya, Ibe Michael Usman, Osuwat Lawrence Obado, Gerald Zirintunda, Fred Ssempijja, Miriam Nansunga, Henry Matovu, Emmanuel Tiyo Ayikobua, Ponsiano Ernest Nganda, David Onanyang, Justine Ekou, Simon Peter Musinguzi, Godfrey Ssimbwa, Keneth Iceland Kasozi

**Affiliations:** ^1^Faculty of Biomedical Sciences, Kampala International University Western Campus, Bushenyi, Uganda; ^2^School of Medicine, Kabale University, Kabale, Uganda; ^3^Department of Anatomy and Cell Biology, Faculty of Health Sciences, Busitema University, Tororo, Uganda; ^4^Graduate Program in Cell Biology, Institute of Biological Sciences, Federal University of Minas Gerais (UFMG), Belo Horizonte, Brazil; ^5^Department of Clinical Pharmacy and Pharmacy Practice, School of Pharmacy, Kampala International University Western Campus, Bushenyi, Uganda; ^6^School of Health Sciences, Soroti University, Soroti, Uganda; ^7^Department of Animal Production and Management, Faculty of Agriculture and Animal Sciences, Busitema University, Tororo, Uganda; ^8^Department of Physiology, College of Health Sciences, Makerere University, Kampala, Uganda; ^9^Department of Biology, Faculty of Science, Gulu University, Gulu, Uganda; ^10^Department of Agriculture Production, Faculty of Agriculture, Kyambogo University, Kampala, Uganda; ^11^Department of Physiology, Faculty of Health Sciences, Muni University, Arua, Uganda

**Keywords:** brain stress, medical education, cerebral cortex, brains in Africans, reaction time (RT), Africa brains, academic stress

## Abstract

**Background:**

Stress among medical students is related to their academic lifespan; however, information on brain health among medical students from developing countries continues to be scarce. The objective of this study was to establish perceived academic stress levels, assess the ability to cope with stress, and investigate its effects on the visual reaction time (VRT), audio reaction time (ART), and tactile reaction time (TRT) in the somatosensory cortex among medical students of Uganda.

**Methods:**

This was a cross-sectional study conducted among preclinical (*n* = 88) and clinical (*n* = 96) undergraduate medical students at Kampala International University Western Campus. A standard Perceived Stress Scale (PSS) was used to categorize stress into low, moderate, and severe while the ability to cope with stress was categorized into below average, average, above average, and superior stresscoper (SS). Data on reaction time were acquired through VRT, ART, and TRT using the catch-a-ruler experiment, and this was analyzed using SPSS version 20.

**Results:**

This study shows that preclinical students are more stressed than clinical students (PSS prevalence for low stress = preclinical; clinical: 40, 60%). Moderate stress was 48.4 and 51.6% while high perceived stress was 75 and 25% among preclinical and clinical students. Among male and female students in preclinical years, higher TRT and VRT were found in clinical students showing that stress affects the tactile and visual cortical areas in the brain, although the VRT scores were only significantly (*P* = 0.0123) poor in male students than female students in biomedical sciences. Also, highly stressed individuals had higher TRT and ART and low VRT. SS had high VRT and ART and low TRT in preclinical students, demonstrating the importance of the visual cortex in stress plasticity. Multiple regression showed a close relationship between PSS, ability to cope with stress, age, and educational level (*P* < 0.05), demonstrating the importance of social and psychological support, especially in the biomedical sciences.

**Conclusion:**

Preclinical students suffer more from stress and are poorer SS than clinical students. This strongly impairs their cortical regions in the brain, thus affecting their academic productivity.

## Introduction

The Perceived Stress Scale (PSS) is a valid tool used for the measurement of stress in humans (1). PSS helps one understand the effects of stress due to its effects on individual confidence and emotion (2). Life-changing events, such as health crises and traumas, can cause depression and anxiety, thus modulating biological processes in affected individuals (3). In academic environments, university students get stressed during the transition into adulthood and independence (4). Therefore, stress has become part of life that directly modulates our general health status (5). When recurring conditions cause stress that is both intense and sustained over a long period, it can be referred to as “chronic” or “toxic” stress (6, 7). In addition, stress when not managed appropriately in its early stages can compromise brain function and general body physiology (8, 9). This is important since stress has been shown to reduce the amygdala in laboratory animals (10); however, epidemiological studies in humans remain scarce. This is important since the amygdala is part of the limbic system which detects stress in the environment, while the prefrontal cortex regulates our reactions to stress (11, 12). In the cerebral cortex, local circuits consist of tens of thousands of neurons, each of which makes thousands of synaptic connections (13). Visual, tactile, and auditory information is processed from the periphery to the cortical level through separate channels that target primary sensory cortices, from which it is further distributed to functionally specialized areas (14, 15). Chronic stress negatively impacts eye and brain function due to an imbalance in sympathetic and vascular function, thus increasing the risk of visual dysfunction (16, 17). Stress is also associated with increased pain disorders (18), and the gate control theory (GCT) of pain has shown a close relationship between pain and touch sensing neurons, i.e., antagonize each other through spinal cord dorsal horn (DH) gating neurons (19, 20). The exact neural circuits underlying the GCT involve a new population of deep layer DH (dDH) inhibitory interneurons that express the receptor tyrosine kinase Ret neonatally (21). Tactile sensation is affected by the stimulus, i.e., stiffness–softness, roughness–smoothness, and tactile comfort scores, which can be evaluated in human (specialists) *via* sensory tests (22). In addition, stress is often associated with auditory phenomena such as hyperacusis, tinnitus, Meniere's disease, and vertigo (23, 24). The auditory and stress systems are linked through the limbic system, which regulates instinctive behavior and emotions *via* the amygdala. In addition, the reticular system, which is responsible for behavior patterns of attention and excitement, projects serotonergic fibers to all pathways of the auditory system, ranging from the cochlea to the auditory cortex (23). Stress effects on the auditory system are less explored, their mechanisms are not well-understood, and recent findings on the functionality of the outer hair cells using distortion product otoacoustic emissions and auditory neurons using evoked auditory brainstem responses showed differences in auditory sensation between different rat strains, i.e., increase and decrease thresholds as would be observed in humans during stress (25). This evidence shows that stress affects the visual, auditory, and tactile cortical areas of the cerebral cortex; however, information on reaction time (RT) in the cerebral cortex is scarce. This is important since recent findings have shown two neurophysiologically distinct and temporally separated integration mechanisms in temporo-parieto-occipital junction and dorsolateral prefrontal cortex and suppression of dominant mechanisms, synthesizing visual and tactile input (26).

During impulse transmission, information moves through the brain (integration center) from the sensory fibers to the motor fibers (27). RT is the time interval between the presentation of an external stimulus and the appropriate voluntary motor response to the stimulus (28). It reflects the speed of the flow of neurophysiological, cognitive, and information processes, which are created by the action of stimulus on the person's sensory system. The receipt of visual, auditory, or tactile information affects processing, decision-making, and time interval for a motor response (28, 29). Stress has been shown to affect mood among college swimming students (30), demonstrating its importance in neuroscience. A recent study reported stress (62%) and burnout (75%) among preclinical medical students (31). In a recent study from Saudi Arabia, the mean age of study participants was 20 years from preclinical and clinical students where the mean PSS was 28.5 ± 3.8 and 59.2% of participants were stressed (32). Stress and burnout among medical students are common problems with likely severe personal and professional effects (33). Globally, numerous studies have reported stress among undergraduate medical students to be 25.6–78% (31); however, studies from Sub-Saharan Africa remain scarce. Excessive stress in medical training predisposes students to difficulties in solving interpersonal conflicts as a result of previous stress (34).

In the psychophysiology field, RT measures are often obtained to index cognitive ability, but a potentially novel use of RTs may be a proxy measure of neural efficiency (35). On a basic level, greater neural efficiency is represented by a smaller variability in RTs or a lower intra-individual mean suggesting that a simple RT computation may not sufficiently capture the cognitive process involved (36), thus the need to assess visual RT (VRT), tactile RT (TRT), and auditory RT (ART). Currently, a chronic scarcity of information relating to stress and RT in stressed medical students in Africa continues to limit progress in neuroscience. This is important since neural education can reduce pain ratings, increase function, address catastrophization, and improve movement in chronic pain (37). In Uganda, RT among medical students is strongly related to the tactile and auditory neural pathways among Ugandans (38); however, information demonstrating the importance of stress among Ugandan medical students and RT created a rationale for this study. This study had the following three objectives: (1) to identify the major stressed medical students and assess their ability to cope with stress; (2) to establish changes in the neural-signaling pathway among preclinical and clinical students of Uganda; and (3) to identify a relationship between RT and stress among medical students of Uganda. Furthermore, limited studies from Sub-Saharan Africa in an academic environment created the need for this study.

## Methods

### Study Design

This was a cross-sectional study carried out at Kampala International University (KIU) Western Campus of South Western Uganda. This study involved medical students who were selected randomly from a class of preclinical and clinical undergraduate students using administrative records from the office of the Dean of Biomedical Sciences and the School of Medicine and Allied Health (KIU). Random purposive sampling was used in this study to select study participants.

#### Inclusion Criteria

Students with no history of academic stress and under normal progress (i.e., without any retakes in the last two semesters) were considered. Students with a healthy physiological background (i.e., no visual disturbance, no audio or hand disability) were included in this study. In this study, only academic-related stress was investigated.

#### Exclusion

Students with psychiatric conditions and with academic-related stress were eliminated from the survey.

#### Sample Size Determination

Students with a history of psychological and mental stress and any major physiological disability were excluded. A total of 184 study participants was chosen using Slovin's formula (39) on sample size as follows:


$$\begin{array}{l} \text{n}=\frac{N}{1+Ne2} \end{array}$$

where *N* = population size, *e* = allowed error of 5%, and *n* = sample size.

### Perceived Stress Determination

This was performed using a standard PSS questionnaire using a 10-point system for diagnosis. Information was then categorized as low stress (LS), moderate stress (MS), and high stressed (HS) individuals with a PSS score of 0–3, a PSS score of 14–26, and a PSS score of 27–40, respectively, as previously determined using a 10-point standard questionnaire (40). In addition, an individual assessment using a 32-point questionnaire was also issued to determine how well individuals cope with stress (https://www.nysut.org/resources/all-listing/2013/april/stress-assessments). The thought control scale, active control scale, social ease scale, tension reduction scale, as well as spiritual practice scales, were determined by adding up specific questions on the 32-point questionnaire. The overall score was obtained by adding up the individual scores and dividing them by 6. The interpretive key was constructed using the following variables, i.e., 3.5 and above were superior stresscopers (SS), 2.5–3.4 were above average stresscopers (AAS), and 1.5–2.4 were suggestive of an average stresscopers (AS), while a score <1.5 was suggestive of a below-average stresscopers (BAS). Prior to the study, the reliability of the tool was tested among 25 students whose data were not included in the analysis. The pretest showed that VRT, TRT, and ART had a Cronbach's alphas of 0.615 from the three items used. The stresscoping 6-point questionnaire had a Cronbach's alphas of 0.60 for use on stresscoping within the study population. The validity of the questionnaire was measured by seeking the views of experts at both the department and faculty level in the field (face validity test) if the tool had its intended purpose (41).

### RT Determination

Individual participants were issued with a structured questionnaire in which they were expected to give responses to their demographics, and 184 healthy undergraduate volunteer students were selected for this study without any visual impairment. All tests were carried out in the Department of Physiology and the Teaching Hospital of Kampala International University. The study environment was quiet, and the temperature was maintained at room temperature. The participants were advised to have an early lunch and allowed 30 min of resting to minimize stress on their bodily performance. VRT, ART, and TRT were determined by the catch-a-ruler experiment with minor modifications. In brief, participants were requested to extend their index finger and thumb, to form a “C” and the ruler (Aim ruler, 30 cm, KEBS, SM#3874, Made in Kenya) was held so that the zero mark was close to the edge of the participant's extended fingers without touching them as previously described (38).

During VRT, the ruler was released randomly within a space of 20 s without making any visual gestures. In ART, the ruler was released after the participant heard the word “hold” being said by the investigator, and their eyes were blindfolded, and no auditory cue was given. Finally, TRT was assessed after tapping the shoulder on the nondominant arm as the ruler was being released. Subsequently, the distance (*d*) of the ruler was recorded in centimeters (cm), and using Newton's 2nd law of motion, RT was calculated using the following formula:


$$\begin{array}{l} T=\surd \left(2^{*}d/981\right). \end{array}$$

Therefore, RT = √2d/981 in seconds.

### Statistical Analysis

Seven values of RT were recorded from each individual during each period of this study with an in-between break of 2 min to eliminate the effect of learning from the participants. The two lowest and two highest values were deleted, and the average of the three values was used to compute the RT for the participant. The data from the VRT, ART, TRT, and PSS were subjected to a D'Agostino & Pearson omnibus normality test for normality testing. The K2 (*P*) values generated were 13.62 (0.0011), 11.89 (0.0026), 19.43 (<0.001), and 0.7012 (0.7043) and subsequently described descriptively using GraphPad prism version 5, and information was described as mean and standard error mean (SEM). Repeated measures of ANOVA were used for analysis using SPSS version 20 statistical software. A *t*-test and Tukey's test were conducted, and a *P* < 0.05 was considered statistically significant.

## Results

### Study Population Demographics

The majority of study participants were males (108/184, 58.7%), were clinical students (96/184, 52.2%), had moderate stress levels (155/184, 84.2%), and moderate stresscopers (168/184, 91.3%). This study also showed that the mean (±SEM) spiritual practice scale scored the highest (3.50 ± 0.04) to support the total population stresscoping score, and the mean age was 23.23 ± 0.28 years (**Table 2**).

### Descriptive Statistics of Stress Among Preclinical and Clinical Students in Uganda

The mean stress score was 18.63 ± 0.43 and 17.31 ± 0.45, while the mean age was 25.81 ± 0.47 years and 20.88 ± 0.10 years in both preclinical and clinical students, respectively (*P* < 0.05). This study showed a low-stress prevalence (25/184) of preclinical and clinical students (40, 60%) among medical students. Moderate stress was 48.4 and 51.6%, while high perceived stress was 75 and 25% among preclinical and clinical students, respectively. The odds of high stress were over four times higher in preclinical than clinical students. The ability to handle stress was three times poorer among preclinical than clinical students while male students were two times more in preclinical than female students (*P* < 0.05). The ability to cope with stress was 3.03 ± 0.03 and 3.07 ± 0.03 among preclinical and clinical students as shown in **Table 3**. Significant observations among preclinical and clinical students were only observed with sex (*P* = 0.006), PSS (*P* = 0.037), and age (0.001).

### Changes in RT Among Preclinical and Clinical Students of Uganda

**Figure 1:**
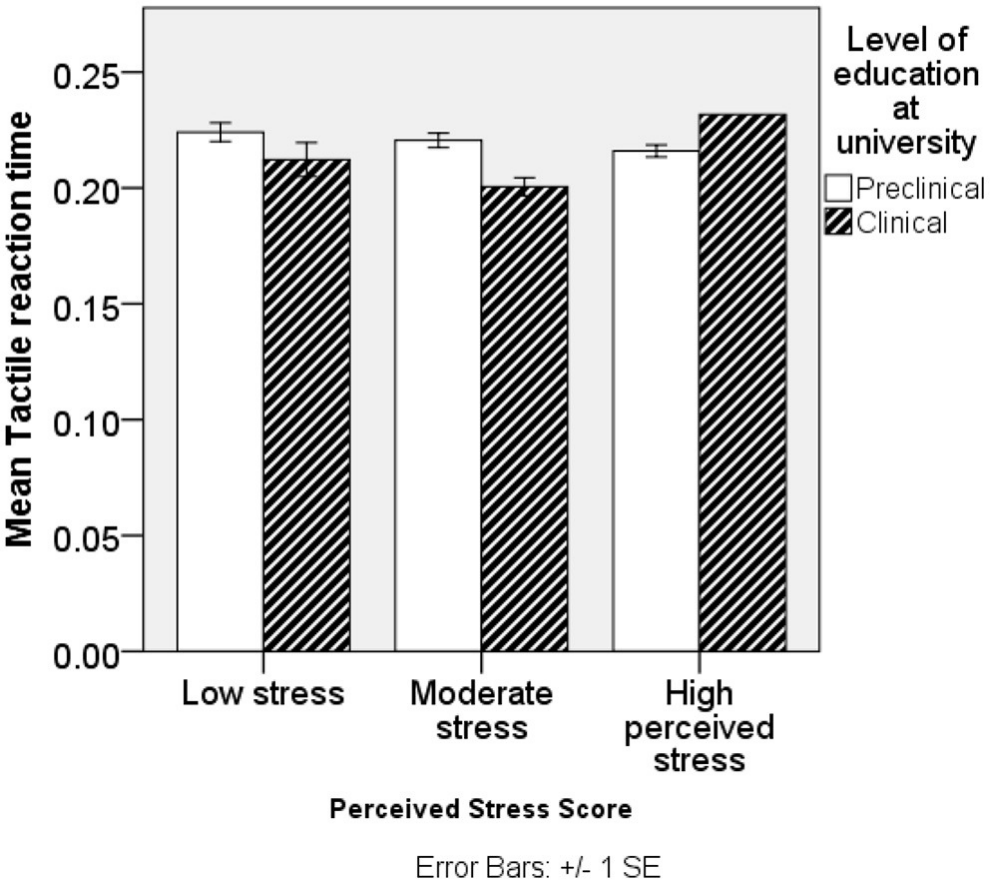
Relationship between perceived stress scores and reaction time among study participants.

The mean RT was higher in preclinical than clinical students on VRT and TRT. VRT < TRT < ART were generally in that order. No significant differences were found in RT of ART, and significant differences in TRT are shown in Figure 1. Among male students, TRT, VRT, and ART were higher among preclinical than clinical students; however, significance was only reported among preclinical students and sex (*P* = 0.0123) with female students having better VRT scores than male students. In female students, TRT and VRT were higher in preclinical than clinical students except for ART. Low and moderate stressed individuals in preclinical had higher TRT, VRT, and ART than their counterparts in clinical students. High-stressed individuals had high TRT and ART, while they had low VRT in preclinical than clinical students. SS had high VRT and ART and low TRT in preclinical students. Above-average stresscopers had high TRT and VRT and low ART in preclinical than clinical students. Average stresscopers had high TRT and VRT and high ART in preclinical than clinical students as shown in **Table 4**. TRT was lowest among clinical students with moderate stress (Figure 1).

### Relationship Between RT and Stress

Simple linear regression showed a low relationship between PSS, RT, and age, i.e., PSS = 15.533 – 0.51 ^*^ ART + 2.776^*^VRT – 10.623 ^*^ TRT + 0.177Age (*R*^2^ = 0.023, ANOVA *P* = 0.373). A multiple regression was run to predict PSS from stress coping scores was conducted, and it was found that PSS = 19.537 – 3.214 × ability to handle stress + 0.789 wellness scale + 1.527 × thought control scale – 1.162 × active coping scale – 0.005 × social ease scale + 0.217 tension reduction scale – 0.045 × spiritual practice scale + 0.078 × age of participant + 0.036 × sex of participant – 0.941 × level of education at university (thought control scale *P* = 0.025 and the rest *P* > 0.05). Logistic regression was performed to determine the effects of education level on thought score, active score, social score, tension score, spiritual score, wellness score, total score, age, sex, ART, VRT, TRT, PSS, and stress coping. The logistic regression model was not statistically significant, *X*^2^ (8) = 1.357, *P* = 0.994. The model explained 84.3% (Negelkerke, *R*^2^) of the variance in the population and correctly classified 91.3% of the participants. The odds ratio of tension score was 33 times higher in preclinical than clinical students (*P* = 0.18), while the odds ratio of age was 0.091 lower in preclinical than clinical students (*P* = 0.000).

### Exploratory Factorial Analysis

The KMO and Bartlett's test [value, χ^2^(d) =0.556, 129.227(10)] showed significant differences (*P* = 0.0001), showing there was no multicollinearity on age, sex of participants, level of education at university, stress diagnosis, and ability to handle stress. This allowed a correlation matrix that identified sex (measures of sampling adequacy (MSA) = 0.745), and this was included in line with Kaiser and Rice's (1974) criteria (42). Eigenvalues for principal component (PC) 1 =1.834 and PC2 = 1.180 were obtained with a cumulative percentage of 60.3%, and PC1 and PC2 had a variance of 95.9 and 1.5%, and after a varimax rotation, the item of participants' ages aligned positively with PC1, while sex aligned positively with PC2. Furthermore, the item level of education aligned negatively with PC1 and PC2, while the ability to handle stress did not align with all components (**Table 5**).

## Discussion

This was the first study reporting stress among medical students in a private institution in Uganda. Heavy enrollment numbers and tuition stress are common in most private universities in Uganda (Tables 1, 2). Generally, students were stressed with a moderate type of stress in agreement with our previous study in the same study population (38). This study showed that stress among medical students was lower than that previously reported among medical students in Saudi Arabia (43). This was important since, in Saudi Arabia, the majority of medical students are male just as it was in this study.

**Table 1:** Sample size determination for study participants.

**Study participants**	**Population size (N) included**	**Sample size (n)**
Preclinical students	113	88
Clinical students	127	96
Total	240	184 (77%)

*This approach was taken to calculate the sample size since we had a defined population and did not have any reference studies in the region to help us use other methods for calculation*.

**Table 2:** Descriptive statistics on social demographics, stress and stress copying levels.

**Variables**		**Statistic**
Sex	Males	108^a^ (58.7^b^)
	Females	76^a^ (41.3^b^)
Education level	Preclinical students	88^a^ (47.8^b^)
	Clinical students	96^a^ (52.2^b^)
Stress diagnosis	Low stress	25^a^ (13.6^b^)
	Moderate stress	155^a^ (84.2^b^)
	High perceived stress	4^a^ (2.2^b^)
Ability to handle stress	Superior stresscopers	11^a^ (6.0^b^)
	Moderate stresscopers	168^a^ (91.3^b^)
	Average stresscopers	5^a^ (2.7^b^)
Wellness scale		3.12 ± 0.03^c^
Thought control scale		3.02 ± 0.04^c^
Active coping scale		2.81 ± 0.03^c^
Social ease scale		2.89 ± 0.03^c^
Tension scale		3.03 ± 0.06^c^
Spiritual practice scale		3.50 ± 0.04^c^
Age of participants (yrs.)		23.23 ± 0.28^c^

a *Frequency,*

b *percentage,*

c *mean ± SEM.*

*SEM, standard error mean*.

The majority of study participants demonstrated moderate stress levels, i.e., 48.4 and 51.6%, in both preclinical and clinical students, respectively (Table 3). Findings in this study demonstrate the importance of stress recognition using heterogeneous sources as long as the subject has sufficient contact with the sensors used to assess the stress (44). Approaches used in this study, though basic, demonstrate novel strategies for adaption in resource-limited settings to promote neural health. Information from this study also showed that medical students in Uganda are less stressed compared to their counterparts in Jedda, Saudi Arabia from which a mean stress score of 28.5 ± 3.8 and prevalence of 59.2% have been reported among medical students (32). Findings in this study justify the use of RT basic experiments in both biomedical and physiatry settings (35).

**Table 3:** Stress, ability to handle stress, and gender among preclinical and clinical students in Uganda.

**Parameter**	**Variable**	**Frequency (%)**			**Odds ratio**	**Test statistic (P-value)**
		**Preclinical**	**Clinical**	**Total**		
Perceived stress	Low stress	10 (40)	15 (60)	25 (100)	1	1.388 (0.239^a^)
	Moderate stress	75 (48.4)	80 (51.6)	155 (100)	1.406	
	High stress	3 (75.0)	1 (25.0)	4 (100)	4.500	
	Total	88 (47.8)	96 (52.2)	184 (100)		
Ability to handle stress	Superior stresscoper	4 (36.4)	7 (63.6)	11 (100)	1	0.886 (0.6511^b^)
	Above average stresscoper	81 (48.2)	87 (51.8)	168 (100)	1.629	
	Average stresscoper	3 (60.0)	2 (40.0)	5 (100)	2.625	
Sex	Male	61 (56.5)	47 (43.5)	108 (100)	2.36	7.850 (0.006^a^)
	Female	27 (35.5)	49 (64.5)	76 (100)		
Perceived stress score	Mean ± SEM	18.63 ± 0.43	17.31 ± 0.45			2.105 (0.037^c^)
Stresscoping	Mean ± SEM	3.03 ± 0.03	3.07 ± 0.03			−0.715 (0.476^c^)
Age	Mean ± SEM	25.81 ± 0.47	20.88 ± 0.10			11.502 (0.001^c^)

*OR, odds ratios. Comparisons made in reference to category 1 during risk estimates.*

a *Chi-square test,*

b *Fisher's exact test,*

c *t-test*.

Preclinical students were more stressed than clinical students, and these had the lowest ability to cope with stress. This was in agreement with a previous study which showed identical results from Saudi Arabia due to stress and burnout (31), showing that medical students in both developing and developed countries suffer from stress while at school. The National Institute of Mental Health defines stress as simply “the brain's response to any demand” (45), and among medical students, academic demands create a lot of emotional stress which would affect their brain function. This was in agreement with findings that medical students have high levels of stress that could be due to daily life stressors, which lead to depression and anxiety (2, 3). The extra stress of academic burden is the breadth and depth of material to be learned, lack of relaxation time, and repeated formative and summative examinations in a competitive environment (46). This calls on policymakers to think critically about the physiological effects of stress among undergraduate medical students in developing countries since stress among undergraduate medical students was 25.6–78% (31). Neurological effects of stress modulate general health (5–9).

The study also showed that RT was in the order of ART > TRT > VRT, and this was higher in preclinical than clinical students (Table 4). RT involves the integration of cerebral cortical regions in the brain, i.e., occipital lobe for VRT, somatosensory area for TRT, and temporal lobe for ART due to specific fibers involved in neural signaling (27, 28). Information from this study shows that the temporal and somatosensory areas of the cerebral cortex among medical students in Uganda were mostly affected by stress. This was important since receipt of visual, auditory, or tactile information affects processing, decision-making, and time interval for a motor response (28–30). In our previous study (38), RT among medical students was found to be in the order of VRT > ART > TRT, and this was lower than that presented in this study. Differences in the study populations are responsible for these changes, this provides evidence to show that RT is plastic (47, 48), and it changes due to environmental factors such as stress. This demonstrates that the plasticity of the cerebral cortex and in stress, the amygdala which detects stress in the environment is reduced in size in laboratory animals (10–12).

**Table 4:** Reaction time among preclinical and clinical students in Uganda.

**Parameter**	**Variable**	**Tactile reaction time (ART)**	**Visual reaction time (VRT)**	**Auditory reaction time (ART)**	**TRT**	**VRT**	**ART**
		**Mean** **±SEM in seconds**			***P*** **value summaries**		
Preclinical	Male	0.223067 ± 0.0027500	0.200618 ± 0.0028075	0.221889 ± 0.0030410	0.2024	0.0123	0.6682
	Female	0.215615 ± 0.0061435	0.197430 ± 0.0043321	0.219511 ± 0.0046744			
Clinical	Male	0.202257 ± 0.0043672	0.200385 ± 0.0061044	0.220438 ± 0.0035438	0.8981	0.3220	0.2605
	Female	0.203149 ± 0.0053535	0.192996 ± 0.0043091	0.225478 ± 0.0027304			
Preclinical	Low stress	0.224080 ± 0.0040541	0.210910 ± 0.0080132	0.221390 ± 0.0065559	HS = 0.880	HS = 0.996	HS = 0.894
	Moderate stress	0.220532 ± 0.0031052	0.197636 ± 0.0024813	0.220833 ± 0.0028452	LS = 0.910	LS = 0.171	LS = 0.997
	High stress	0.216000 ± 0.0026000	0.212167 ± 0.0060842	0.228533 ± 0.0073295	MS = 0.951	MS = 0.495	MS = 0.850
Clinical	Low stress	0.212233 ± 0.0073521	0.203540 ± 0.0066102	0.221353 ± 0.0068263	HS = 0.578	HS = 0.963	HS = 0.650
	Moderate stress	0.200565 ± 0.0038718	0.195206 ± 0.0042734	0.223213 ± 0.0023663	LS = 0.223	LS = 0.420	LS = 0.765
	High stress	0.231700	0.205300	0.231700	MS = 0.362	MS = 0.785	MS = 0.703
Preclinical	Superior stresscoper	0.200075 ± 0.0204689	0.185725 ± 0.0105357	0.227300 ± 0.0073780	AS = 0.666	AS = 0.996	AS = 0.994
	Above average stresscoper	0.221315 ± 0.0027122	0.200342 ± 0.0024484	0.220559 ± 0.0027071	SS = 0.226	SS = 0.403	SS = 0.847
	Average stresscoper	0.233967 ± 0.0065920	0.199233 ± 0.0142653	0.229167 ± 0.0113943	AAS = 0.183	AAS = 0.703	AAS = 0.815
Clinical	Superior stresscoper	0.213843 ± 0.0040066	0.178771 ± 0.0104802	0.224700 ± 0.0078991	AS = 0.930	AS = 0.835	AS = 0.992
	Above average stresscoper	0.201789 ± 0.0037631	0.198075 ± 0.0039743	0.222786 ± 0.0023765	SS = 0.641	SS = 0.371	SS = 0.973
	Average stresscoper	0.203950 ± 0.0233500	0.195500 ± 0.0130000	0.226850 ± 0.0134500	AAS = 0.996	AAS = 0.995	AAS = 0.964

*Unpaired t-tests were conducted on sex. Multivariate analysis (Tukey's test P-values) conducted on stress diagnosis and stress coping. The horizontal variable was compared against HS, MS, and LS. HS, high stress; MS, moderate stress; LS, low stress. Tukey's test compares the horizontal variables against stresscoping on AS, SS, and AAS, i.e., average stresscopers, superior stresscopers, and above-average stresscopers, respectively*.

The ability to cope with stress among Ugandan medical students was found to be above average, and this was associated with low TRT, VRT, and ART in clinical students (Table 4). This is the first study from a developing country demonstrating the importance of the temporal lobe in stress, although previous studies have been conducted in developed countries (35, 44). The efferent auditory pathways modulate outer hair cells of the cochlea, protect against noise, and improve the detection of sound sources in noisy environments, and female smokers have been associated with higher auditory disorders than men due to DNA damage (49). Average stresscopers had TRT > VRT > ART and ART > TRT > VRT among preclinical and clinical students, respectively. This showed that stress effects in preclinical students are mainly associated with the somatosensory area of the cerebral cortex. A new population of deep neurons receives excitatory and inhibitory inputs on tactile stimuli, and this directly interacts with tactile and pain polysynaptic pathways (19–21). Low TRT is important for clinical students since they have to learn how to respond to tactile sensations. These stimuli can be affected by the stiffness–softness and roughness–smoothness (22), thus of major importance during clinical examination of the nervous system in clinical students.

Stress effects on the auditory system are less explored, their mechanisms are not well-understood, and recent findings on the functionality of the outer hair cells using distortion product otoacoustic emissions and auditory neurons using evoked auditory brainstem responses showed differences in auditory sensation between different rat strains, i.e., increased and decreased thresholds as would be observed in humans during stress (25). This evidence shows that stress affects the visual, auditory, and tactile cortical areas of the cerebral cortex; however, information on RT in the cerebral cortex is scarce. This is important since recent findings have shown two neurophysiologically distinct and temporally separated integration mechanisms in temporo-parieto-occipital junction and dorsolateral prefrontal cortex and suppression of dominant mechanisms synthesizing visual and tactile input (26).

Finally, multiple regression analysis showed a close relationship between PSS scores and major stress coping variables, age, and level of education (*P* < 0.05). This was important since stress is modulated by the neural environment which affects neural health (50). Logistic regression showed that the tension scale was significantly different among preclinical and clinical students. This showed that the inability to handle stress would lead to the development of tension among preclinical students, thus affecting neural development in Ugandan medical students. No direct relationships were found between PC1 (age) and PC2 (sex), showing that findings in the study (Table 5) cut across the study population. Strategies to promote psychiatry support across all medical students would help improve students' physiological status.

**Table 5:** Rotated component matrix^a^ and component covariance correlation after varimax rotation.

	**Raw** **Component**		**Rescaled** **Component**		**Component** **correlation**
	**1**	**2**	**1**	**2**	
Age of participant	3.664		0.984		
Level of education at university	−0.327		−0.652		*r = 0.00*
Stress diagnosis					
Ability to handle stress					
Sex of participant		0.490		0.993	

*Extraction method: principal component analysis. Rotation Method: Varimax with Kaiser Normalization.*

a *Rotation converged in three iterations*.

## Conclusion

In this study, we demonstrated that the VRT is affected independently across the gender. These findings, though basic, highlight challenges that medical students encounter especially during their critical biomedical years while at medical school. We reported poor adaptability to stress in our study population demonstrating a need for psychological counseling to help students adapt easily to their hectic medical career since this has direct implications on the somatosensory pathways which are crucial during their academic lifecycle. We also recognized challenges associated with the relatively small sample size, thus prospective studies could be conducted involving large populations to help improve knowledge in this field among Sub-Saharan African medical students since this could help to increase the reliability of the statistical tests further. We also recommend further studies to assess psychiatric stress levels among undergraduate students since this was not investigated in this study.

## Data Availability Statement

The datasets presented in this study can be found in online repositories. The names of the repository/repositories and accession number(s) can be found below: https://figshare.com/s/b1fd222cf4d295ed0d9d.

## Ethics Statement

The studies involving human participants were reviewed and approved by the Kampala International University Ethics Review Board under number BSCPHY/7792/163/DU. The patients/participants provided their written informed consent to participate in this study.

## Author Contributions

KIK conceptualized and designed the study and drafted the initial manuscript. RM, JJO, AMA, RN, IE, KM, OHA, GN, IMU, OLO, ETA, PEN, and GS collected the data. KIK and RM conducted the statistical analysis. MK, HIN, EDE, SOS, EK, IE, AOO, KM, OHA, IMU, OLO, GZ, FS, MN, SPM, and GS interpreted the data. All authors reviewed and approved it for publication.

## Conflict of Interest

The authors declare that the research was conducted in the absence of any commercial or financial relationships that could be construed as a potential conflict of interest.

## Publisher's Note

All claims expressed in this article are solely those of the authors and do not necessarily represent those of their affiliated organizations, or those of the publisher, the editors and the reviewers. Any product that may be evaluated in this article, or claim that may be made by its manufacturer, is not guaranteed or endorsed by the publisher.
